# 地榆皂苷II通过ITGB4/FAK信号通路抑制非小细胞肺癌的进展

**DOI:** 10.3779/j.issn.1009-3419.2026.102.10

**Published:** 2026-04-20

**Authors:** Jiaxuan REN, Yani LIU, Bo WANG, Jiayi LV, Yunbo DI, Zhenwen CHEN, Yirong XU

**Affiliations:** ^1^030000 太原，山西医科大学研究生学院; ^1^Graduate School, Shanxi Medical University, Taiyuan 030000, China; ^2^032200 汾阳，山西医药学院基础医学部; ^2^Department of Basic Medicine, Shanxi University of Medicine, Fenyang 032200, China; ^3^032200 汾阳，山西省汾阳医院病理科; ^3^Department of Pathology, Shanxi Province Fenyang Hospital, Fenyang 032200, China

**Keywords:** 肺肿瘤, 地榆皂苷II, ITGB4/FAK信号通路, 进展, Lung neoplasms, Ziyuglycoside II, ITGB4/FAK pathway, Progression

## Abstract

**背景与目的** 非小细胞肺癌（non-small cell lung cancer, NSCLC）的发病率和死亡率总体呈现上升趋势，患者整体预后不佳，现有临床治疗手段存在明显的局限性。而中药生物活性成分具有作用靶点多样、毒副作用小的独特优势。地榆皂苷II（ziyuglycoside II, ZGS II）是地榆的一种主要活性成分，可抑制癌细胞的增殖、迁移以及侵袭过程，这一特性使其在癌症治疗领域展现出了潜在的应用价值和发展前景。然而，ZGS II对NSCLC的作用及其可能的机制仍未阐明。本研究旨在探讨ZGS II在NSCLC中抗肿瘤的分子机制，探讨作为改善NSCLC患者预后的一种新的治疗策略的可能性。**方法** 首先采用细胞增殖与毒性检测试剂盒（cell counting kit-8, CCK-8）和集落形成实验检测ZGS II处理NSCLC细胞后活力和增殖能力的变化。细胞划痕和Transwell实验评估ZGS II对NSCLC细胞的迁移和侵袭能力的影响。利用蛋白印迹法、慢病毒转染、细胞划痕和Transwell等方法研究ZGS II对整合素β4/黏着斑激酶（integrin β4/focal adhesion kinase, ITGB4/FAK）信号通路的作用。随后使用FAK激活剂ZINC40099027进行逆转实验。采用裸鼠异种移植模型评价ZGS II对体内NSCLC转移的抑制作用。**结果** ZGS II抑制NSCLC细胞的增殖、迁移和侵袭，并且抑制了ITGB4的蛋白表达，这一结果在敲低ITGB4后抑制作用明显加强。同时发现总FAK的表达几乎不变，但磷酸化黏着斑激酶（phosphorylated focal adhesion kinase, p-FAK）的蛋白表达水平受到抑制。加入FAK激活剂后，细胞的迁移率以及穿过Transwell膜的细胞数量增加，ZGS II对细胞迁移和侵袭能力的抑制作用得到逆转。因此，ZGS II的抗肿瘤作用依赖于ITGB4/FAK信号通路的激活减弱。裸鼠异种移植实验结果表明，ZGS II显著抑制了裸鼠体内肿瘤的生长。**结论** ZGS II通过抑制ITGB4/FAK通路抑制NSCLC的增殖、转移和肿瘤生长。这些结果突出了ZGS II作为治疗转移性NSCLC的药物的前景，为其临床研究奠定基础。

## Abstract (en)

**Background and objective** The incidence and mortality rates of non-small cell lung cancer (NSCLC) are generally on the rise, and the overall prognosis of patients remains poor. Current clinical treatment strategies have significant limitations. Notably, the bioactive components of traditional Chinese medicine possess unique advantages, including diverse targets of action and low toxicity. Ziyuglycoside II (ZGS II), a major active component of Sanguisorba officinalis L, has been shown to inhibit cancer cell proliferation, migration, and invasion. This property confers potential application value and developmental prospects for its use in cancer therapy. Nevertheless, the effects of ZGS II on NSCLC and the underlying mechanisms remain unclear. This study aimed to investigate the anti-tumor molecular mechanism of ZGS II in NSCLC and to explore the potential of being a novel therapeutic strategy for improving the prognosis of NSCLC patients. **Methods** First, the cell counting kit-8 (CCK-8) and the colony formation assay were used to detect the changes in the viability and proliferation ability of NSCLC cells after treatment with ZGS II. Wound-healing and Transwell assays were used to evaluate the effects of ZGS II on migration and invasion abilities. Western blot, Lentiviral transfection, wound healing assay, and Transwell assay were used to study the effects of ZGS II on the integrin β4/focal adhesion kinase (ITGB4/FAK) signaling pathway. A rescue experiment was then performed using the FAK agonist ZINC40099027. A nude mouse xenograft model was used to evaluate the inhibitory effect of ZGS II on NSCLC metastasis *in vivo*. **Results** ZGS II inhibited proliferation, migration, and invasion of NSCLC cells and suppressed the protein expression of ITGB4. This inhibitory effect was significantly enhanced upon ITGB4 knockdown. Meanwhile, total FAK expression remained nearly unchanged, but the protein expression level of phosphorylated focal adhesion kinase (p-FAK) was suppressed. Following the addition of an FAK activator, cell migration rate and the number of cells crossing the Transwell membrane increased, reversing the inhibitory effects of ZGS II on cell migration and invasion. Therefore, the anti-tumor effect of ZGS II depends on the reduced activation of the ITGB4/FAK signaling pathway. The nude mouse xenograft experiment results showed that ZGS II effectively inhibited tumor growth in nude mice. **Conclusion** ZGS II inhibits proliferation, metastasis, and tumor growth in NSCLC by suppressing the ITGB4/FAK pathway. These findings highlight the potential of ZGS II as a promising drug for treating metastatic NSCLC and lay the foundation for its clinical research.

肺癌是严重威胁人类生命健康的疾病之一，其发病率及死亡率均居恶性肿瘤首位。其中非小细胞肺癌（non-small cell lung cancer, NSCLC）占全球肺癌病例的80%-85%，NSCLC又可分为腺癌、鳞癌和大细胞癌。NSCLC的早期症状不明显，5年生存率较低^[1]^。常规干预手段往往疗效有限，难以实现理想的治疗结局。对于早期患者，手术切除是优先选择的治疗手段；放化疗具有较强的毒副作用，会给患者身体带来沉重负担，部分患者因难以耐受而不得不中断治疗；靶向治疗是针对肿瘤细胞特定的治疗靶点进行治疗，疗效较好且副作用相对较小，但只适用于有相应治疗靶点的患者；免疫检查点抑制剂治疗中程序性细胞死亡配体1（programmed cell death ligand 1, PD-L1）表达是最常用的生物标志物，对部分PD-L1表达阳性的患者有效，但标志物较少，不够完善，并且还容易产生耐药性^[2]^。因此迫切需要开发新的，危害更小的、更加有效的治疗方法，从而潜在地减少转移性扩散并改善患者的预后。

地榆药材是地榆或者长叶地榆的干燥根。其中以地榆皂苷II（ziyuglycoside II, ZGS II）为代表的三萜类化合物是地榆中重要的生物活性成分之一。研究^[3]^表明，ZGS II可以有效改善放化疗造成的外周血细胞减少症状，还具有抗炎、抗氧化的作用。ZGS II在各种类型的恶性癌症中显示出抗肿瘤作用。如：ZGS II可抑制结直肠癌、骨肉瘤、肝癌等癌细胞的增殖和转移^[4-6]^。利用网络药理学探讨地榆的治疗潜能，结果表明地榆对肺肿瘤、乳腺肿瘤、肝细胞癌等多种肿瘤有较好的治疗潜能，并发现ZGS II抑制肺腺癌细胞的增殖。然而，ZGS II在NSCLC中抗肿瘤的具体机制，尚未阐明。

在ZGS II治疗肺腺癌的关键靶点中发现了一个有潜力的靶点：整合素β4（integrin β4, ITGB4）。ITGB4在各类恶性肿瘤组织中表达量显著上调，并且这种高表达与患者预后不良有显著关联。ITGB4作为黏着斑激酶（focal adhesion kinase, FAK）的上游调控因子，影响其表达和磷酸化^[7]^。例如：SPC25通过ITGB4促进肝癌细胞的转移，沉默ITGB4后FAK、PI3K、AKT的磷酸化均降低^[8]^。ITGB4/FAK信号通路在癌细胞转移相关的信号级联反应中至关重要。例如：Bufotalin通过调控ITGB4/FAK信号通路从而诱导胶质母细胞瘤细胞的凋亡^[9]^；ZGS II通过抑制ITGB4/FAK信号通路从而抑制乳腺癌MDA-MB-231细胞的转移^[10]^。因此，ITGB4/FAK信号通路在恶性肿瘤的进展中发挥着重要作用，本研究旨在探讨ZGS II是否通过调控该通路发挥抗肿瘤效应，为ZGS II应用于NSCLC的治疗提供理论依据。

## 1 材料与方法

### 1.1 材料

ZGS II（纯度：99.77%，批号：35286-59-0）购自MedChemExpress公司。DME/F-12 1:1（1×）（批号：AL30835590）和RPMI Medium Modified培养基（批号：AL30842761）购自Cytiva公司。胎牛血清（批号：2503101）购自Viva Cell Biosciences公司。青霉素-链霉素-两性霉素B混合溶液（批号：2500080007）、RIPA裂解液（批号：240011009）、BCA蛋白浓度测定试剂盒（批号：2500080014）、二甲基亚砜（批号：34241216013）均购于Solarbio公司。胰蛋白酶（批号：MA0232-Nov-06K）购自Meilunbio公司。Transwell细胞小室（批号：4015018）购买于康宁Falcon。Matrigel基质胶（批号：WC6407500）购于Yeasen公司。CCK-8溶液（批号：20C11B60）和超敏ECL化学发光即用型底物（批号：19H09A71）购自BOSTER公司。PrimeScript^TM^ RT Master Mix（批号：A011061A）和TB Green^®^ Premix Ex Taq^TM^ II（批号：A010474A）购自Takara公司。本研究使用的抗体如下：ITGB4（A01015-2, BOSTER）（批号：236777F21），GAPDH（A19056, ABclonal）（批号：3507443006），FAK（12636-1-AP, Proteintech）（批号：00177971），P-FAK（83933-1-RR, Proteintech）（批号：23013606）。

### 1.2 细胞培养

人NSCLC细胞系：A549（目录号：SCSP-503）和NCI-H226（目录号：SCSP-5073）细胞购自中国科学院细胞库。A549细胞在DME/F-12 1:1（1×）培养基中培养，NCI-H226在RPMI Medium Modified培养基中培养，2种培养基中均添加10%胎牛血清和1%青霉素-链霉素-两性霉素B混合溶液，2种细胞均置于37 ^o^C、含5% CO_2_的细胞培养箱内进行培养。

### 1.3 细胞活力测定

用CCK-8法测定细胞活力。将A549和NCI-H226细胞分别按照5×10^3^个细胞/每孔的密度接种到96孔板中，培养24 h，然后用浓度梯度为0、1、5、10、20、40、80 µmol/L的ZGS II分别处理24和48 h。随后按照CCK-8溶液:培养基=1:10的比例配置成CCK-8工作液，在细胞培养箱中孵育2 h，用SpectraMax^®^ Absorbance Reader酶标仪检测450 nm处的吸光度（A）值。细胞的生长抑制效应用以下公式计算：细胞存活率（%）=（A_处理组_/A_对照组_）× 100%。

### 1.4 克隆形成实验

将A549和NCI-H226细胞按照1×10^4^个细胞/每孔分别接种于6孔板中，用不同浓度的ZGS II处理7-14 d，每隔3 d更换新的完全培养基。直至克隆形成，PBS洗涤，4%多聚甲醛固定，0.1%结晶紫染色液染色，采集图像。

### 1.5 细胞划痕实验

将A549和NCI-H226细胞分别按照2.5×10^5^个细胞/每孔接种到6孔板中，待细胞铺展密度达80%左右，使用200 μL移液枪头沿垂直方向制造划痕，PBS溶液洗涤3次。然后加入含药物的无血清培养基混合液孵育24 h，用倒置显微镜拍摄0及24 h的划痕照片，用Image J软件测量划痕距离来评估细胞迁移能力。迁移率（%）=（0 h划痕距离-24 h划痕距离）/0 h划痕距离×100%。

### 1.6 Transwell迁移和侵袭实验

将含2.5×10^5^个细胞和ZGS II的200 μL的无血清培养基加入上室（孔径为8 μm）进行迁移实验，侵袭实验需要提前加入基质胶。这两种实验中均需要在下室中加入500 μL含20%胎牛血清的培养基，随后在5% CO_2_、37 ^o^C的培养箱中继续培养24 h。Transwell上室经过4%多聚甲醛固定30 min，0.1%结晶紫染色15 min，PBS冲洗3次，用棉签擦去未穿过小室膜的细胞，用倒置显微镜随机选取5个不重叠的视野拍照。

### 1.7 蛋白印迹法

将被处理后的细胞加入含蛋白酶和磷酸酶抑制剂的RPMI裂解液中裂解30 min，4 ^o^C下120,000 rpm离心20 min后收集上清液。借助BCA蛋白浓度测定试剂盒来测定各样本的蛋白浓度。把提取的总蛋白置于8%SDS-PAGE凝胶上进行分离，再转移至PVDF膜上。用含5%脱脂奶粉的TBST封闭液封闭2 h，随后将PVDF膜在4 ^o^C条件下与特异性抗体（ITGB4: 1:1000; GAPDH: 1:20,000; FAK: 1:20,000; P-FAK: 1:1000）孵育过夜。用TBST缓冲液洗膜3次，每次5 min，随后把膜放入含有以1:5000比例稀释的辣根过氧化物酶偶联二抗的溶液中孵育2 h，TBST洗膜，增强化学发光试剂进行膜显影。

### 1.8 逆转录实时荧光定量聚合酶链式反应（reverse transcription quantitative polymerase chain reaction, RT-qPCR）

TRIzol^®^试剂提取总RNA，用PrimeScript^TM^ RT Master Mix试剂合成cDNA，qPCR的热循环条件为：初始变性（95 ^o^C、60 s），40个循环步骤（95 ^o^C、20 s，60 ^o^C退火20 s，72 ^o^C延伸30 s）。然后使用TB Green^®^ Premix Ex Taq^TM^ II定量ITGB4和GAPDH的表达水平。qPCR使用的引物为：GAPDH：F-5′-AGAACATCATCCCTGCCTCTACTGG-3′，R-5′-GACGCCTGCTTCACCACCTTC-3′；ITGB4，F-5′-TCGCTCTACACGGACACCATCTG-3′，R-5′-TCC ACCATCTTGACCTTGAAGTTGC-3′。采用2^-ΔΔCt^方法定量mRNA表达。

### 1.9 细胞转染

为了敲低ITGB4基因的表达，按照试剂商的说明书，使用转染试剂转染ITGB4 shRNA（序列：GGAAGGTCACCAACAACATGC），scramble shRNA（序列：TTCTCCGAACGTGTCACGT）作为对照组，慢病毒合成由吉凯公司提供。将A549和NCI-H226细胞按照5×10^4^个细胞/每孔分别接种于6孔板中，37 ^o^C培养至细胞汇合度为20%-30%，加入含慢病毒和感染增强液的混合液，感染8-16 h后，将原培养基更换为完全培养基，待感染72 h后观察感染效果。为了构建敲低表达稳定的细胞系，将细胞继续培养于含2 µg/mL嘌呤霉素的培养液中筛选48 h后收集细胞进行功能验证。

### 1.10 免疫组织化学技术

用4%多聚甲醛固定液固定肿瘤组织，石蜡包埋，切片。70 ^o^C烤片2 h，二甲苯脱蜡，逐级酒精水化，然后1%过氧化氢避光孵育1 h，将切片放入pH 9.0的EDTA抗原修复液中用微波炉修复15 min，待其冷却后，用10%马血清封闭1 h，兔单克隆一抗放入湿盒中4 ^o^C孵育过夜，检测ITGB4、FAK、p-FAK（稀释比列为1:100）的表达情况。PBS洗涤3次后，用酶标山羊抗小鼠/兔IgG聚合物37 ^o^C孵育切片30 min。二氨基联苯胺四盐酸盐显色，自来水终止后苏木素染色15 min，快速蘸取分化液，氨水反蓝，最后逐级酒精脱水，二甲苯透明，中性树脂封片。

### 1.11 裸鼠NSCLC模型

雄性BALB/c-nu小鼠（*n*=10，5周龄，16-18 g）构建异种移植瘤模型。裸鼠饲养于长期维持在（21±2）^o^C、45%±10%湿度和24 h光照/黑暗循环的环境中，严格遵循动物福利原则。将裸鼠随机分为2组（*n*=5），选择对数生长期的敲低ITGB4和未敲低ITGB4的A549细胞，调整细胞密度为2.5×10^6^个细胞/100 μL，PBS重悬，置于冰上低温保存，在裸鼠右侧腋窝皮下尽快完成注射，然后每隔3 d观察肿瘤的生长情况。在体内实验中，根据参考文献选择10 mg/kg的给药剂量，在该浓度下显示出了抗肿瘤活性并且没有毒性。按照2% DMSO储备液+40% PEG300+5% Tween-80+53%生理盐水的比例配置10 mg/kg ZGS II，根据裸鼠每日体重计算给药体积量，每隔1 d腹腔注射，连续30 d，处死动物。收集肿瘤组织及主要脏器，放入4%多聚甲醛溶液中固定，脱水后将组织包埋于石蜡盒中进行组织病理学分析。裸鼠许可证编号：SYXK（晋）2024-0007，实验项目伦理编号：2025082。

### 1.12 统计分析

采用GraphPad Prism软件（San Diego, California USA）进行统计分析。所有数据结果以均数±标准差（Mean±SD）表示。*t*检验用于两组之间的比较，*P*<0.05为差异具有统计学意义。

## 2 结果

### 2.1 ZGS II抑制NSCLC细胞的活力及增殖能力

为了研究ZGS II对人NSCLC细胞活力的影响，将A549和NCI-H226细胞暴露于浓度逐渐增加的ZGS II中24和48 h后，A549细胞的活力呈现出剂量和时间依赖性下降，NCI-H226细胞的活力呈现出剂量依赖性下降。给药24 h后，A549和NCI-H226细胞的半数抑制浓度（half inhibitory concentration, IC_50_）分别为33.41和13.35 μmol/L；给药48 h后A549和NCI-H226细胞的IC_50_分别为28.22和12.76 μmol/L（图1A）。克隆形成实验显示，与0 μmol/L组相比，A549细胞在16 μmol/L ZGS II处理组的集落形成明显减少，NCI-H226细胞在6 μmol/L ZGS II处理组的集落形成也明显减少，差异具有统计学意义，进一步证实了ZGS II对NSCLC细胞的增殖具有明显的抑制作用（图1B）。以上结果表明ZGS II具有抑制细胞的活力及增殖能力。

**图1:**
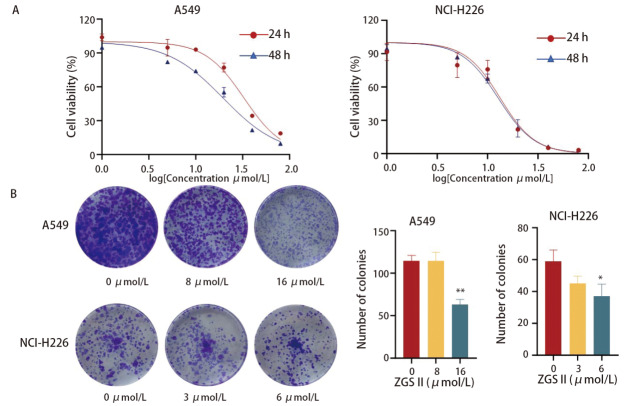
ZGS II抑制NSCLC细胞活力及增殖能力。A：CCK-8法检测A549和NCI-H226细胞的细胞活力；B：克隆形成实验检测ZGS II对A549和NCI-H226细胞增殖的影响。与0 *μ*mol/L组相比，*P<0.05，**P<0.01。

### 2.2 ZGS II抑制人NSCLC细胞在体外的迁移和侵袭

细胞划痕实验表明，随着药物浓度的升高，NSCLC细胞的迁移率下降，细胞的迁移能力下降（图2A、2B）。Transwell迁移实验表明，细胞穿过Transwell膜的数量随着药物浓度的升高而减少，ZGS II抑制了NSCLC细胞的迁移（图3A）。用基质胶包被Transwell小室进行侵袭实验证实了ZGS II对NSCLC细胞侵袭的抑制作用，显示突破基质胶的细胞数量减少（图3B）。这些发现表明，ZGS II显著限制了NSCLC细胞的迁移和侵袭能力。

**图2:**
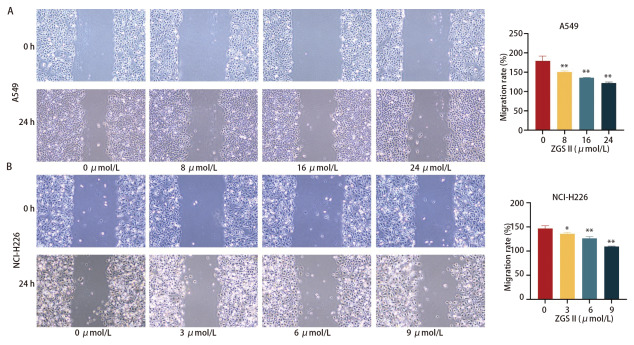
细胞划痕实验检测ZGS II对A549细胞（A）和NCI-H226细胞（B）迁移能力的影响。与0 *μ*mol/L组相比，*P<0.05，**P<0.01。

**图3:**
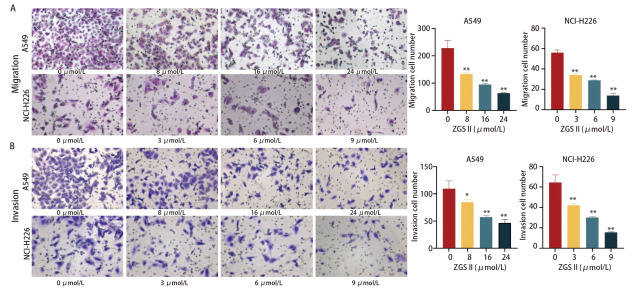
Transwell实验检测ZGS II对A549和NCI-H226细胞迁移能力（A）和侵袭能力（B）的影响（×100）。与0 *μ*mol/L组相比，*P<0.05，**P<0.01。

### 2.3 ZGS II通过ITGB4/FAK信号通路抑制细胞的迁移和侵袭

在A549细胞中，随着ZGS II浓度升高，ITGB4的表达水平呈现出下降趋势，并在16和24 μmol/L ZGS II处理后差异明显，具有统计学意义。FAK作为ITGB4的下游调控蛋白，结果表明FAK总蛋白的表达量虽然没有变化，但p-FAK在24 μmol/L时表达明显降低（图4A）。为了进一步阐明ITGB4在ZGS II抑制NSCLC细胞进展中的作用，在A549和NCI-H226细胞中转染了ITGB4 shRNA，结果表明敲低ITGB4后蛋白和mRNA水平均降低，且差异具有统计学意义，表明敲低模型构建成功（图4B-4F）。在A549细胞中，与ITGB4-NC组相比，加入ZGS II处理后细胞的迁移率下降，在敲低ITGB4后迁移率下降更加明显（图5A）。在NCI-H226细胞中呈现出同样的趋势（图5B）。Transwell迁移和侵袭实验表明，与ITGB4-NC组相比，加入ZGS II处理后穿过Transwell膜的细胞数量减少，这一结果在敲低ITGB4后穿过Transwell膜的细胞数量显著减少，显著抑制细胞的迁移和侵袭能力（图6A-6B）。这些结果说明ZGS II可以通过ITGB4/FAK通路抑制NSCLC细胞的迁移和侵袭。

**图4:**
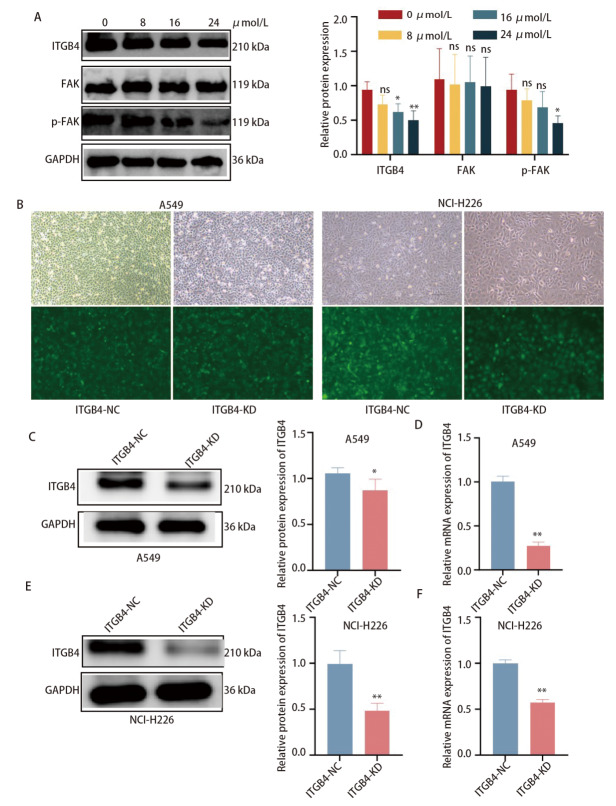
ZGS II处理NSCLC细胞对ITGB4/FAK表达水平的影响。A：Western blot实验检测ZGS II处理后ITGB4、FAK、p-FAK的蛋白表达水平；B：A549和NCI-H226细胞的慢病毒敲低ITGB4的荧光图（×40）；C：Western blot实验检测A549细胞中ITGB4的蛋白表达水平；D：RT-qPCR实验检测A549细胞中ITGB4的mRNA表达水平；E：Western blot实验检测NCI-H226细胞中ITGB4的蛋白表达水平；F：RT-qPCR实验检测NCI-H226细胞中ITGB4的mRNA表达水平。与ITGB4-NC组相比，*P<0.05，**P<0.01。

**图5:**
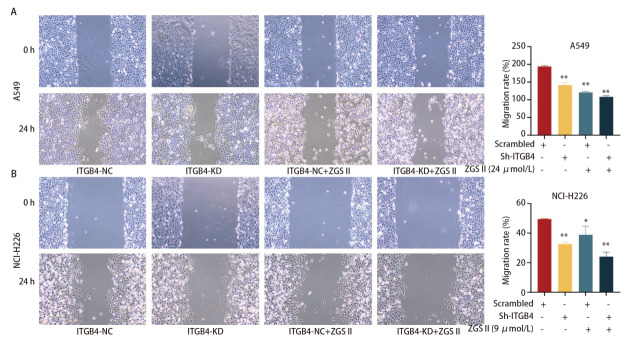
细胞划痕实验检测ZGS II通过ITGB4/FAK通路对A549细胞（A）和NCI-H226细胞（B）迁移能力的影响。与ITGB4-NC组相比，*P<0.05，**P<0.01。

**图6:**
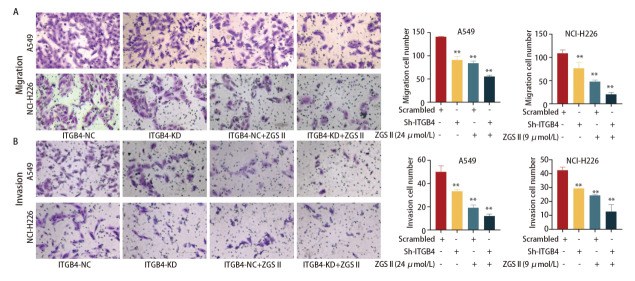
Transwell实验检测ZGS II通过ITGB4/FAK通路对A549和NCI-H226细胞迁移能力（A）和侵袭能力（B）的影响（×100）。与ITGB4-NC组相比，*P<0.05，**P<0.01。

### 2.4 FAK激活剂逆转ZGS II对NSCLC细胞迁移和侵袭的抑制

使用FAK激活剂ZINC40099027（ZINC）激活FAK的活性，在A549细胞中，与对照组相比，加入ZGS II处理后细胞迁移能力下降；加入ZINC处理后细胞迁移能力增加，但没有统计学意义。与ZGS II单独处理相比，加入ZINC后细胞迁移能力增加（图7A）。在NCI-H226细胞中出现同样的趋势，但与对照组相比，单独加入ZINC处理后细胞划痕迁移能力增加，具有统计学意义（图7B）。在Transwell迁移和侵袭实验中也得到了同样的结果，与对照组相比，ZGS II处理后穿过Transwell膜的细胞数量减少；加入ZINC后穿过Transwell膜的细胞数量增多。与ZGS II单独处理后相比，加入ZINC处理后穿过Transwell膜细胞数目增多，细胞的迁移和侵袭能力增加（图8A、2B）。以上结果说明，添加FAK激活剂后ZGS II对NSCLC细胞的迁移和侵袭的抑制作用得到逆转，更进一步证明ZGS II可以通过ITGB4/FAK信号通路发挥抗肿瘤的分子机制。

**图7:**
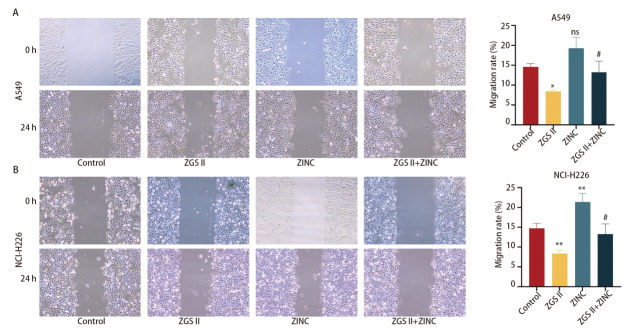
细胞划痕实验检测在A549细胞（A）和NCI-H226细胞（B）中加入FAK激动剂后对细胞迁移能力的影响。与对照组相比，*P<0.05，**P<0.01；与ZGS II组相比，^#^*P*<0.05。

**图8:**
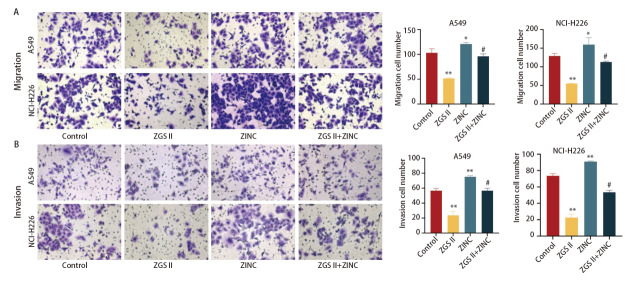
Transwell实验检测在A549和NCI-H226细胞中加入FAK激动剂后对细胞迁移能力（A）和侵袭能力（B）的影响（×100）。与对照组相比，*P<0.05，**P<0.01；与ZGS II组相比，^#^*P*<0.05。

### 2.5 ZGS II通过ITGB4/FAK信号通路减弱NSCLC的体内的进展

为了研究ZGS II通过ITGB4/FAK途径抑制在体内的抗肿瘤作用，将未敲低ITGB4和敲低ITGB4的A549细胞分别注射到裸鼠右侧腋窝皮下。成瘤后，在小鼠腹腔内注射ZGS II，剂量为10 mg/kg，每隔1 d注射1次，连续30 d（图9A）。结果显示，ZGS II处理后，敲低ITGB4显著抑制了肿瘤的生长（图9B-9D），ITGB4的蛋白和mRNA表达水平均降低（图9E、9F）。免疫组化结果表明，ZGS II处理后，敲低ITGB4，ITGB4的表达水平更低，但FAK、p-FAK的表达水平几乎没有发生变化，提示ZGS II主要通过抑制ITGB4表达间接削弱下游蛋白FAK的活化状态，而非影响FAK总蛋白本身的合成与降解（图9G）。ZGS II通过ITGB4/FAK通路抑制肿瘤生长。值得注意的是，ZGS II处理后心、肝、脾、肾等主要脏器的组织病理学检查未见不良的病理性改变，说明ZGS II没有毒性（图9H）。

**图9:**
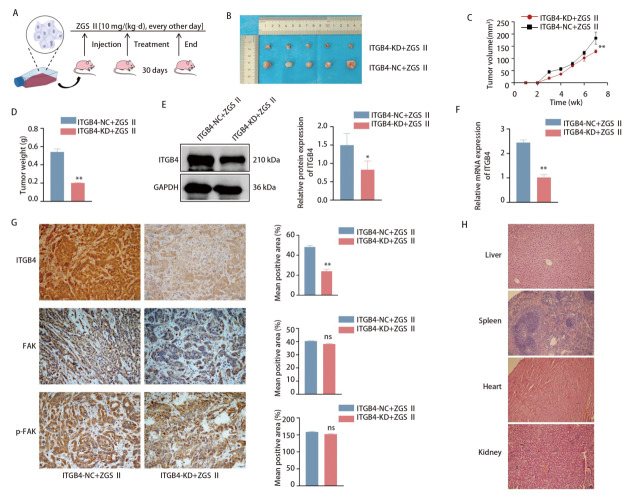
ZGS II通过调节ITGB4/FAK信号通路减弱NSCLC的体内进展。A：动物实验流程图；B：裸鼠移植瘤大体图；C：裸鼠肿瘤体积统计图；D：裸鼠肿瘤重量统计图；E: Western blot实验检测移植瘤中ITGB4蛋白表达水平的条带图和统计图；F：RT-qPCR实验验证移植瘤中ITGB4的mRNA表达水平统计图；G: 免疫组化实验检测移植瘤中ITGB4、FAK、p-FAK的表达水平（×200）；H：裸鼠心、肝、脾、肾的HE染色（×100）。与ITGB4-NC+ZGS II组相比，*P<0.05，**P<0.01。

## 3 讨论

NSCLC患者发现时往往分期较晚而且可选择的治疗方法有限，预后效果较差。放化疗、靶向治疗和免疫检查点抑制剂等治疗方法在临床应用中面临诸多问题，开发新的安全有效的治疗方法是解决当前癌症治疗困境的关键举措^[11]^。已证实中药生物活性成分具有抗肿瘤作用。ZGS II是从地榆中提取出的三萜类化合物，具有抗炎、抗氧化和抗肿瘤作用。研究^[12]^表明ZGS II抑制多种恶性肿瘤的增殖、迁移和侵袭，并且可以通过低氧诱导因子-1α（hypoxia inducible factor-1α, HIF-1α）/糖酵解途径抑制A549和H1299细胞的迁移和侵袭，但该研究主要围绕肿瘤代谢方面进行研究。本研究聚焦ZGS II在NSCLC转移机制展开，为其对NSCLC抗肿瘤进展方面提供了一定的理论依据。

为了研究ZGS II是否对NSCLC有抑制作用，在本研究中，首先检测ZGS II对NSCLC细胞的细胞毒性，发现随着药物浓度升高，NSCLC细胞的活力呈现出剂量和时间依赖性下降。克隆形成、划痕和Transwell实验结果有力表明，ZGS II抑制细胞的增殖、迁移和侵袭。随后我们采用裸鼠体内实验进一步验证了上述结果，证明ZGS II可以抑制肺癌移植瘤的生长。因此ZGS II具有明显抑制肿瘤生长的作用，可以成为一种潜在发挥肿瘤抑制作用的中药制剂。

网络药理学分析表明，ITGB4、MMP9和BCL2等多个基因可能是ZGS II治疗肺腺癌的靶点；并且有研究^[10]^表明，ZGS II处理三阴性乳腺癌细胞后，ITGB4及p-FAK的表达水平下降，抑制ITGB4/FAK信号通路的激活从而抑制细胞的迁移和侵袭。提示ZGS II可能是通过ITGB4这一靶点发挥抑癌作用。ITGB4作为整合素家族的一员，在细胞的黏附过程、恶性进展、转移扩散以及信号传导等生物学活动中扮演着关键角色，并且在多种恶性肿瘤组织中高表达，与患者预后情况呈现明显负相关，因此ITGB4是一种在癌症患者中具有预测和预后价值的分子标志物^[13]^。本研究结果表明，ZGS II处理后ITGB4水平降低，同时显著抑制了细胞的迁移和侵袭能力，这一结果在敲低ITGB4后抑制作用明显加强，因此更加印证了ZGS II可以通过抑制ITGB4发挥抑癌作用。

FAK是一种非受体型酪氨酸激酶，是细胞增殖、迁移、侵袭以及耐药的关键信号中枢，作为ITGB4的下游调控因子，参与了多种癌症的生长和进展^[14]^。如在膀胱癌中，FN1可以通过ITGB4/FAK信号通路抑制膀胱癌细胞的凋亡，从而增加化疗的耐药性^[15]^；在胃癌中，ITGB4可以激活FAK促进SOX2的表达，进而促进胃癌细胞的转移^[16]^。本研究结果发现，ZGS II处理后，FAK总蛋白虽然不变，但p-FAK的表达呈现下降趋势。以上结果表明ZGS II可能通过抑制ITGB4/FAK通路发挥抑制NSCLC迁移和侵袭的作用。

随后为了进一步验证，使用FAK激活剂ZINC40099027激活FAK进行逆转实验。ZINC40099027是一种特异性的FAK激活剂，促进FAK的磷酸化，但它不激活其旁系同源物Pyk2和Src。ZINC是一种直接且高效的FAK激活剂^[17]^。在本研究中，使用ZGS II并同时加入ZINC后细胞迁移和侵袭能力上升，从而证明ZINC逆转了ZGS II对NSCLC细胞迁移和侵袭的抑制作用，本研究与王琳等^[18]^的研究报道结果一致，该研究表明，ZINC逆转梓醇对结肠癌细胞迁移和侵袭的抑制作用。因此，ZGS II通过ITGB4激活FAK发挥抑制NSCLC的进展。除此之外，有研究^[10]^表明，EGFR与ITGB4相互作用，当EGFR被激活时发生自身的磷酸化，进而激活ITGB4，ITGB4进一步激活其下游FAK，FAK随后激活AKT和p38MAPK信号通路，发挥抑制肿瘤生长和转移的作用。

综上所述，在本研究中，ZGS II抑制了NSCLC细胞的增殖、迁移和侵袭，同时ZGS II通过ITGB4/FAK信号通路发挥抗肿瘤作用，为ZGS II治疗NSCLC提供了初步的理论依据。

然而，本研究仍存在一定局限性：本实验仅选取肺腺癌A549细胞与肺鳞癌NCI-H226细胞两种经典肺癌细胞系开展研究，未在多株不同来源的肺癌细胞系中验证结果。后续将扩大细胞系范围，进一步验证ZGS II对肿瘤的抑制作用。其次，ZGS II抑制了ITGB4的表达，但由于ITGB4基因的编码序列较长，增加了载体的构建难度，而且目前没有针对ITGB4的激动剂，因此本研究未对ITGB4进行过表达干预研究，仅干预了下游分子FAK。最后，本研究尚未开展肿瘤转移相关体内实验，暂未验证ZGS II对体内肿瘤转移的调控作用。后续将构建肿瘤转移模型进行体内研究，进一步探究ZGS II用于转移性NSCLC治疗的应用潜力。

另外中药活性成分具有多靶点、广谱、无毒的优点，但是单药难以达到治疗效果，临床往往与放化疗联合使用，作为一种新型的抗肿瘤中药成分，在NSCLC中ZGS II是否可以与化疗、靶向治疗和免疫治疗等多种治疗方法联合应用，从而提高治疗效果，这将是我们下一步要探索的内容。
